# An integrated primary care approach for frail community-dwelling older persons: a step forward in improving the quality of care

**DOI:** 10.1186/s12913-017-2827-6

**Published:** 2018-01-17

**Authors:** Lotte Vestjens, Jane M. Cramm, Anna P. Nieboer

**Affiliations:** Erasmus School of Health Policy & Management, Erasmus University Rotterdam, P.O. Box 1738, 3000 DR Rotterdam, the Netherlands

**Keywords:** Integrated care, Quality of primary care, Chronic care model, Frailty, Elderly, Healthcare professionals, Mixed methods

## Abstract

**Background:**

High-quality care delivery for frail older persons, many of whom have multiple complex needs, is among the greatest challenges faced by healthcare systems today. The Chronic Care Model (CCM) may guide quality improvement efforts for primary care delivery to frail older populations. Objectives of this study were to assess the implementation of interventions in CCM dimensions, and to investigate the quality of primary care as perceived by healthcare professionals, in practices following the Finding and Follow-up of Frail older persons (FFF) integrated care approach and those providing usual care.

**Methods:**

Structured interviews were conducted with general practitioners (GPs) from 11 intervention practices and 4 control practices to assess the implementation of interventions. A longitudinal survey (12-month period, 2 measurement timepoints) was conducted to assess the quality of primary care as perceived by healthcare professionals (intervention and control GP practices) using the Assessment of Chronic Illness Care Short version (ACIC-S). Independent-samples *t*-tests were used to assess differences in ACIC-S scores between groups. Interviews were conducted with GPs from the intervention practices to gain a deeper understanding of their experiences with the FFF approach.

**Results:**

Intervention practices implemented significantly more interventions congruent with (dimensions of) the CCM compared with control GP practices. With respect to the quality of primary care as perceived by healthcare professionals, mean ACIC-S scores for all CCM dimensions and overall mean ACIC-S scores were significantly higher in the intervention group than in the control group at the follow-up timepoint. The number of implemented interventions was associated positively with perceived quality of primary care (ACIC-S scores) at follow-up. Important motives of GPs to implement the FFF approach were the aging of the population and transformations in the primary care sector. Proactive care delivery and multidisciplinary collaboration were considered to be essential. Major challenges to the implementation and embedding of the FFF approach were structural financing and manpower, and the availability of a facilitating information and communication technology system.

**Conclusions:**

Our study showed that proactive, integrated care that is based on (elements of) the CCM may be a step forward in improving quality of care for frail older persons.

## Background

Increasing age and increasing level of frailty tend to go together [1, 2]. Frailty refers to a dynamic state that affects an older adult who experiences problems or losses in several domains of human functioning (physical, psychological, and social domains) [3]. Frail older people have substantially increased risks of disability, institutionalization, multimorbidity, and mortality [2, 4–8]. The healthcare needs of community-living frail older people are often multifaceted and complex. In addition, the co-occurrence of frailty, disability, and/or multimorbidity increases the complexity of older patients’ healthcare needs and the need for high-quality care [8].

High-quality care delivery for frail older persons, many of whom have multiple complex needs, is one of the greatest challenges faced by healthcare systems [9, 10]. In the Netherlands, care for frail older adults is increasingly being delivered in a primary care setting, with gatekeeping general practitioners (GPs) at the core of the system [11, 12]. However, current primary healthcare systems are ill equipped to meet long-term complex healthcare needs of frail older persons, given that primary care services are predominantly fragmented, reactive, and disease oriented [13, 14].

In response to the challenges posed by the growing complexity of patients’ healthcare needs, models of integrated care delivery have emerged. Integrated care is increasingly being advocated as a means to improve quality of care and patient outcomes for community-dwelling frail older patients [10, 15]. Integrated care can be defined as “a well planned and well organized set of services and care processes, targeted at the multi-dimensional needs/problems of an individual client, or a category of people with similar needs/problems” ([16], p. 18). Integrated care approaches need to be patient-centered, which can be achieved by establishing partnerships between older patients and healthcare professionals who work together to optimize patient outcomes [13]. The delivery of effective and high-quality integrated primary care for frail community-living older patients requires fundamental and comprehensive changes to the design of practice [17]. To guide quality improvement efforts in primary care delivery, Wagner and colleagues [17–20] developed the Chronic Care Model (CCM). The CCM is based on the premise that high-quality care and improved patient outcomes result from the provision of proactive, patient-centered, integrated care [21]. It entails six interrelated key system elements for the provision of effective care in primary care practices: (1) self-management support, (2) delivery system design, (3) decision support, (4) clinical information systems, (5) the healthcare system, and (6) the community. Ongoing self-management support (1) needs to be provided to frail older patients by (teams of) professionals. This process involves the collaborative assistance of frail older patients in acquiring the necessary knowledge, skills, and confidence to self-manage their health and well-being successfully. A well-designed proactive delivery system (2) facilitates effective, efficient care and self-management support. It requires, for example, a well-functioning team of professionals, planned patient interactions, regular follow-up, and case management for patients with complex needs. To deliver optimal care to frail older persons, evidence-based guidelines should be embedded in daily practice through reminders and feedback. Moreover, specialist expertise needs to be incorporated in primary care (3). Clinical information systems (4) need to facilitate communication among involved healthcare professionals and the delivery of effective care by providing reminders, sharing information, monitoring performance, and organizing patient-related data. These primary care-based components reside in the broader healthcare system (5), which in turn is embedded in the larger community (6), with all of its resources and policies [17, 19, 22].

Many studies have assessed the effectiveness of care programs that are based on the CCM. For example, Coleman, Austin, Brach, and Wagner [23] reviewed evidence of the CCM’s effectiveness for a diverse range of patients in primary care practice. In general, care that is congruent with dimensions of the CCM can lead to improved care delivery and better patient outcomes. Changes in practices falling within the scope of multiple components of the CCM have been associated with better care quality. However, most studies have focused on patients with specific chronic conditions, such as diabetes and asthma [23]. Studies involving broader populations of older patients, without focusing on particular chronic conditions, are limited [24].

We aimed to increase our knowledge about CCM implementation for frail older persons in the primary care setting and to assess the quality of proactive, integrated primary care. We thus comparatively assessed a proactive, integrated care program and usual primary care for community-living frail older persons. Our first objective was to examine the implementation of interventions in the six areas of system redesign proposed by the CCM, i.e., linkages to community resources, organization of healthcare, self-management support, delivery system design, decision support, and clinical information systems. We assessed the congruency of primary care with (elements of) the CCM in the practices of GPs who implemented a proactive, integrated care program and those delivering usual primary care. Second, we aimed to investigate the quality of primary care as perceived by healthcare professionals involved in care delivery in these settings.

In the present study, we evaluated the “Finding and Follow-up of Frail older persons” (FFF) program, which aims to improve the quality of care and well-being of frail community-dwelling persons aged 75 years and older. The proactive FFF approach to integrated care was implemented in several GP practices in the western part of North Brabant Province, the Netherlands, to effectively redesign the fragmented and reactive primary care system. Its ultimate goals are to meet the long-term, complex healthcare needs and preferences of frail older adults and to improve their well-being. The FFF approach combines multiple interrelated and promising components that are assumed to encourage the provision of high-quality integrated primary care to frail older persons, such as proactive case finding, case management, medication review, self-management support, and multidisciplinary teamwork. These interrelated key components are combined in a comprehensive integrated primary care approach which is expected to improve quality of primary care, and ultimately to influence older patients’ well-being.

## Methods

### Study design and setting

The present study is part of a large-scale evaluation of the effectiveness of the FFF approach in improving the quality of primary care and older persons’ well-being. It was conducted in the western part of North Brabant Province, the Netherlands. The evaluation study had a quasi-experimental design and was performed between 2014 and 2017. GP practices were considered to be eligible for participation in the intervention group of the study if they recently implemented the FFF approach and were not involved in other research projects. GP practices were considered eligible for participation in the control group if they were not engaged in proactively screening for frailty among their older patient population yet. In addition, GP practices that already follow-up older persons in a systematic way were not considered to be eligible to participate as control practices. We approached 17 GP practices for participation in this study (12 intervention practices and 5 control practices). In total, 11 GP practices that implemented the FFF approach (intervention group) and 4 GP practices that provided primary care as usual (control group) participated in the evaluation. The study protocol was reviewed by the medical ethics committee of the Erasmus Medical Centre in Rotterdam, the Netherlands (study protocol number MEC-2014-444). The committee decided that the rules laid down in the Medical Research Involving Human Subjects Act did not apply (for a detailed study protocol, see Vestjens, Cramm, Birnie, and Nieboer: Evaluating an integrated primary care approach to improve well-being among frail community-living older people: a theory-guided study protocol, submitted).

The present study had a mixed-methods (quantitative and qualitative) design. To assess the quality of primary care and gain a deeper understanding of experiences with proactive integrated care, we examined the implementation of interventions falling under the scope of the CCM dimensions in participating GP practices. We collected qualitative data in face-to-face interviews with GPs from practices providing care according to the FFF approach, and carried out a longitudinal questionnaire survey among healthcare professionals to assess the quality of primary care in FFF and usual care practices.

### Quality of primary care: Implementation of interventions falling under CCM dimensions

In structured interviews with the 11 participating GPs from FFF practices, conducted in 2015, we assessed exactly how care was delivered and which interventions were implemented successfully. We also assessed the provision of usual care to community-dwelling older patients by conducting structured interviews with the 4 GPs from control practices in 2015. All interviews were conducted at the GPs’ practices using a template based on the six areas of system redesign proposed in the CCM [17–19, 22]. The interview template was initially developed for the assessment of interventions implemented in disease management programs for chronically ill patients [25]. It was adjusted to include important interventions related to primary care delivery for frail older patients. All interventions were classified according to the six areas of system change in the interview format (Table 1). Lincoln and Guba [26] argue that ensuring credibility is one of the most important aspects in establishing trustworthiness when it comes to qualitative research. This means that the specific procedures employed, such as the line of questioning pursued in the data gathering sessions and the methods of data analysis, should be derived, where possible, from those that have been successfully utilized in previous comparable projects [27]. Therefore we used a template based on the six areas which has already been successfully used before [25].

**Table 1:** Overview of interventions implemented in intervention (FFF approach) and control (usual primary care) GP practices

CCM dimension	Intervention	Intervention practices (*n* = 11)		Control practices (*n* = 4)	
		*n*	%	*n*	%
Healthcare organization	Integrated financing	2	18	0	0
Healthcare organization	Specific policies and subsidies for immigrant population	0	0	0	0
Healthcare organization	Sustainable financing agreements with health insurers	4	36	0	0
Healthcare organization	Financing Geriatric Care Module	10	91	0	0
Community linkages	Multidisciplinary and transmural collaboration	3	27	1	25
Community linkages	Shared structural approach between hospital and primary care	3	27	2	50
Community linkages	Setting up transmural care pathways/care protocols	3	27	2	50
Community linkages	Referral and information exchange arrangements between primary and hospital care	5	45	3	75
Community linkages	Cooperation with external community partners	11	100	4	100
Community linkages	Joint treatment plan between primary and hospital care	3	27	1	25
Community linkages	Involvement of patient groups and panels in care design	0	0	0	0
Community linkages	Communication platform between stakeholders about patients	2	18	0	0
Community linkages	Role model in the area	5	45	0	0
Community linkages	Regional training course	9	82	2	50
Community linkages	Regional collaboration for the care of frail older persons	8	73	1	25
Community linkages	Family participation	11	100	4	100
Community linkages	Geriatric network	1	9	0	0
Self-management support	Promotion of disease-specific information	11	100	3	75
Self-management support	Individual care plan	10	91	2	50
Self-management support	Diagnosis and treatment of mental health issues	10	91	3	75
Self-management support	Lifestyle intervention (e.g., physical activity, diet, smoking)	8	73	2	50
Self-management support	Support of self-management (e.g., Internet)	5	45	3	75
Self-management support	Telemonitoring	1	9	0	0
Self-management support	Personal coaching	10	91	4	100
Self-management support	Motivational interviewing	6	55	1	25
Self-management support	Reflection interviews	0	0	0	0
Self-management support	Informational meetings	2	18	0	0
Self-management support	Group session for patient and family	1	9	0	0
Self-management support	Cognitive behavioral therapy	3	27	2	50
Decision support	Care standards/clinical guidelines	11	100	4	100
Decision support	Uniform treatment protocol in outpatient and inpatient care	2	18	1	25
Decision support	Training and independence of practice nurses	9	82	3	75
Decision support	Professional education and training for care providers	9	82	3	75
Decision support	Audit and feedback	4	36	1	25
Decision support	Use of care protocols for immigrants	0	0	0	0
Decision support	Structural participation in knowledge exchange/best practices	3	27	0	0
Decision support	Quality of life questionnaire	7	64	1	25
Decision support	Automatic measurement of process/outcome indicators	3	27	1	25
Decision support	Evaluation of healthcare via focus groups with patients	0	0	1	25
Decision support	Measurement of patient satisfaction	5	45	2	50
Decision support	Guideline Finding and Follow-up of Frail older persons	10	91	0	0
Decision support	Guideline Geriatric Care Module	11	100	0	0
Delivery system design	Delegation of care from GP to (practice) nurse	9	82	2	50
Delivery system design	Substitution of inpatient with outpatient care	8	73	2	50
Delivery system design	Intensifying collaboration with ongoing projects	6	55	2	50
Delivery system design	Systematic follow-up of patients	9	82	2	50
Delivery system design	Specific plan for immigrant population	0	0	0	0
Delivery system design	Joint Medical Consult	1	9	0	0
Delivery system design	Meetings of professionals from different disciplines to exchange information	11	100	2	50
Delivery system design	Joint consultations	0	0	0	0
Delivery system design	Proactive monitoring of high-risk patients	11	100	1	25
Delivery system design	Board of clients	0	0	0	0
Delivery system design	Bottleneck analysis between professionals and patients	0	0	0	0
Delivery system design	Stepped care method	4	36	0	0
Delivery system design	Expansion of chain of care to the secondary care setting	3	27	1	25
Delivery system design	Proactive screening for frailty	11	100	0	0
Delivery system design	Medication review	11	100	3	75
Clinical information systems	Electronic patient records system with patient portal	3	27	1	25
Clinical information systems	GP information system	11	100	4	100
Clinical information systems	Chain information system (e.g., COPD, diabetes)	11	100	4	100
Clinical information systems	Use of ICT for internal and/or regional benchmarking relevant for frail older patients	4	36	0	0
Clinical information systems	Systematic registration by every caregiver	9	82	3	75
Clinical information systems	Creation of a safe environment for data exchange	8	73	4	100
Clinical information systems	Exchange of information among care disciplines	8	73	3	75
Average number of interventions implemented		33		23	

*COPD* Chronic Obstructive Pulmonary Disease, *FFF* Finding and Follow-up of Frail older persons, *GP* general practitioner, *ICT* information and communication technology

During interviews, all GPs (*n* = 15) were asked to indicate which interventions falling within the scope of the CCM dimensions were implemented in their practices. GPs were also allowed to mention and add interventions that were not included in the interview format. All interviews were approximately 60–75 min in length and were recorded with permission of the GPs. Altogether, an extensive description of implemented interventions was retrieved.

### Quality of primary care, as perceived by healthcare professionals

#### Longitudinal survey

The longitudinal survey study involved two measurement timepoints to enable detection of potential differences over a 12-month period. At baseline (T0; autumn 2014), a questionnaire was sent to all 112 professionals involved in care provision at participating intervention and control GP practices. A total of 75 healthcare professionals (57 in the intervention group and 18 in the control group) completed the questionnaire (67% response rate). One year later (T1; autumn 2015 and beginning of 2016), we approached all 108 professionals who were (still) involved in care provision at the participating practices. A total of 78 healthcare professionals (55 in the intervention group, 23 in the control group) completed the questionnaire at T1 (72.2% response rate). Some responding professionals in the intervention group, such as elderly care physicians, were involved simultaneously in several of the intervention GP practices.

Healthcare professionals were asked to complete the Assessment of Chronic Illness Care Short version (ACIC-S) [28]. This comprehensive instrument focuses on the organization of healthcare, rather than conventional outcome measures or process indicators [29]. The ACIC-S is based on the six areas of system change advocated by the CCM to affect the quality of healthcare: linkages to community resources, organization of healthcare, self-management support, delivery system design, decision support, and clinical information systems [17–19, 22]. The questionnaire is composed of three items per area, which represent a continuum from poor to optimal organization and support of CCM-based care delivery. Participants were asked to indicate the degree of implementation of each component on a four-point scale ranging from “little or no implementation” to “fully implemented.” For example, for the “linkages to community resources” area, little or no implementation suggests that partnerships with community organizations do not exist and full implementation is in place when such partnerships are actively sought to develop formal supportive programs and policies throughout the entire system. Within each of the four levels of implementation, participants were asked to rate the degree to which the description applied on a three-point scale. The resulting scale ranged from 0 to 11, with categories defined as little or no support (0–2), basic or intermediate support (3–5), advanced support (6–8), and optimal or comprehensive integrated care (9–11) [28, 29]. We derived subscale scores for individual CCM dimensions by calculating the average of the three item scores. Subscale scores were derived when responses for at least two of the three items were available. Total scores were calculated by averaging subscale scores when at least four of six such scores were available. Cronbach’s alpha values for the ACIC-S were 0.90 at T0 and 0.93 at T1.

#### Statistical analyses

Descriptive statistics were used to characterize the population of healthcare professionals in the control and intervention groups. We used independent-samples *t*-tests and chi-squared tests to investigate differences between groups. Independent-samples *t*-tests were used to assess differences between interventions and control practices regarding the aggregated mean number of interventions implemented in both groups. Correlation analysis was used to assess the association between the number of interventions implemented and the perceived quality of primary care. Results were considered statistically significant when two-sided *p*-values were <0.05.

#### Qualitative interviews

In addition to the structured interviews with GPs to assess the implementation of interventions, we interviewed the 11 GPs from intervention practices extensively to provide a deeper and richer understanding of their experiences with the FFF approach in their practices. Subjects central to the interviews were: (1) motives for FFF approach implementation, (2) differences between the FFF approach and usual care and among intervention GP practices, and (3) challenges related to the implementation and embedding of the FFF approach. GPs were encouraged to discuss their experiences in detail, and allowed to introduce new subjects. These face-to-face interviews were conducted at the GPs’ practices and recorded with their permission.

#### Analysis of qualitative interview data

Latent content analysis [30, 31], which focuses primarily on the underlying meaning of content [32], was used to examine qualitative interview data. Interview texts were in Dutch and were translated into English during the writing of the report. All interview texts were read multiple times to gain a holistic understanding. Meaning units were extracted, coded, and categorized. Underlying meanings of categories were expressed in themes [30]. The results were presented by interview subject.

## Results

### Motives of GPs in the intervention group to implement the FFF approach

Interviews with the GPs in the intervention group revealed that the aging of the population makes the implementation of proactive, integrated care delivery, as in the FFF approach, important. They explained that their patient population shows an evident increase in the proportion of community-living (frail) older persons with often complex (healthcare) needs. Moreover, GPs emphasized that the transformation of the healthcare sector is an important reason to redesign primary care delivery for older adults and improve the quality of primary care. GPs mentioned that enabling older persons to live independently in the community for as long as possible is the avowed ambition of policy makers. They noted a shift toward more primary and community care: “Especially the changes in the healthcare sector are important. Nursing homes are closing. We sat together with two other colleagues from three GP practices. We can do two things: we can wait and see what happens or we can anticipate.” These were the most important motives of GPs to implement the FFF approach.

### Implementation of interventions in the intervention and control GP practices

Table 1 shows the interventions implemented in the intervention and control GP practices according to CCM dimension. On average, more interventions that were in line with the CCM were implemented in intervention than in control GP practices (*n* = 33 (range, 23–42) vs. *n* = 23 (range, 14–33)). This difference was significant (*p* = 0.014; *n* = 15). Intervention GP practices redesigned their care delivery and processes when considering the implementation of interventions related to the FFF approach. More such interventions (e.g., use of individualized care plans, delegation of care from GPs to (practice) nurses, systematic follow-up of patients, meetings of professionals in different disciplines to exchange information, proactive monitoring of high-risk patients, proactive screening for frailty, and medication reviews) were implemented in intervention than in control GP practices.

### Differences between the FFF approach and usual care, as experienced by GPs

GPs providing care according to the FFF approach considered proactive care delivery (e.g., monitoring of high-risk patients and screening for frailty) and multidisciplinary collaboration (e.g., meetings of professionals from different disciplines and delegation of care from GPs to (practice) nurses) to be particularly important. The majority of GPs indicated that the traditional primary care system for (frail) older persons was mostly reactive and fragmented, and did not enable effective coping with the complex (healthcare) needs of community-dwelling older patients: “Especially when it is very busy in the GP practice there is a risk of providing reactive care, while at this moment [with the FFF approach] you are forced to deliver proactive care and anticipate.” GPs indicated that proactive care and case finding of frail community-dwelling older persons could minimize acute (health) problems and promote the use of preventive care in some cases. The majority of GPs considered multidisciplinary collaboration, including multidisciplinary consultation, to be important. Participants stated that multidisciplinary collaboration can, for example, enhance the expertise of involved professionals and promote a holistic view of an older person’s (complex) health problems and demands: “It is good that someone else is involved too, an elderly care physician for example. It is easier to consult others. A specialist’s viewpoint can be included.” Some GPs indicated that care can be tailored to the needs and wishes of patients and that more attention can be paid to frail older patients. Several GPs also explained that case managers had important coordinating roles in the care process.

### Variation among intervention GP practices, as experienced by GPs

Interviews revealed that GPs also observed differences among intervention GP practices with regard to the implementation and execution of (elements of) the FFF approach. We mention the most important of these differences. First, although all GPs used the same screening instrument to identify frailty among community-living older adults, the selection of patients prioritized for screening differed among practices. For example, several GPs indicated that they selected older patients based on gut feelings, i.e., a “sense of alarm,” whereas others explained that they prioritized patients who had no regular contact with professionals in their practices. Moreover, the (number of) professionals involved in frailty screening differed among GP practices. Whereas homecare, geriatric, and practice nurses screened for frailty in some practices, professionals from only one of these disciplines performed screening in others. Second, aspects of multidisciplinary consultation, such as frequency, the number of older patients discussed, and the professionals involved, differed among GP practices. One important difference was the degree of professionals’ involvement in social care, which ranged from close collaboration to non-involvement in multidisciplinary consultation and care for frail older patients. Finally, GPs considered that the guidelines on the long-term follow-up of frail older persons were not comprehensive enough. Differences existed with respect to who served the lead role and the organization of follow-up. The training of professionals focused mainly on screening procedures, with little addressing of the long-term follow-up of frail older adults. One GP reported non-use of individualized care plans to report plans and actions, which were reported only in the practice’s information system.

### Quality of primary care, as perceived by healthcare professionals

In addition to the interviews held with GPs, we used a longitudinal questionnaire survey to assess perceived quality of primary care among all healthcare professionals in the intervention and control practices. Here, we report results concerning the quality of primary care, as assessed using the ACIC-S.

#### Baseline characteristics of healthcare professionals

Table 2 shows the baseline characteristics of healthcare professionals in the intervention and control groups. At T0, 57 healthcare professionals in the intervention group completed the questionnaire. This group consisted of GPs (21.1%), homecare nurses (15.8%), case managers and geriatric nurses (15.8%), GP assistants (8.8%), practice nurses (7.0%), physiotherapists (7.0%), occupational therapists (7.0%), elderly care physicians (5.3%), and other professionals (e.g., social workers and dieticians; 12.2%). The mean age of these professionals was 42.6 years; almost 81% of them were female and nearly 95% had high educational levels (higher professional education or university). Almost 65% of professionals in the intervention group had worked at their organizations for at least 3 years, and more than 84% worked at least 22 h per week. Eighteen healthcare professionals in the control group completed the questionnaire at T0. This group consisted of GPs (33.3%), GP assistants (27.8%), practice nurses (16.7%), physiotherapists (5.6%), homecare nurses (5.6%), dieticians (5.6%), and other professionals (5.4%). The mean age of control professionals was 44.7 years; nearly 78% of them were female and more than 72% had high educational levels. More than 83% of these professionals had worked in their organizations for at least 3 years, and nearly 78% worked at least 22 h per week. The percentages of healthcare professionals with high educational levels differed significantly between the intervention and control groups (chi-squared test, *p* < 0.05; Table 2).

**Table 2:** Characteristics of healthcare professionals at baseline

Characteristic	Control group (*n* = 18)	Intervention group (*n* = 57)
	*n* (%) or mean (SD)	*n* (%) or mean (SD)
Age (years)	44.72 (12.39)	42.60 (11.38)
Gender (female)	14 (77.8%)	46 (80.7%)
Educational level (high)	13 (72.2%)	54 (94.7%)*
Working in organization (≥ 3 years)	15 (83.3%)	37 (64.9%)
Working hours (≥22 h per week)	14 (77.8%)	48 (84.2%)

No value is missing in either group. *SD* standard deviation. *p < 0.05 (two-tailed), independent-samples *t*-test and chi-squared test

Table 3 shows ACIC-S scores at T0 and T1. Average baseline scores in the control group ranged from 3.78 (standard deviation (SD) = 2.31) for the healthcare organization dimension to 6.18 (SD = 2.28) for the clinical information systems dimension. The overall mean baseline ACIC-S score in the control group was 5.26 (SD = 1.61), indicating basic or intermediate support for integrated care for frail older persons. Average baseline scores in the intervention group ranged from 5.54 (SD = 1.68) for the decision support dimension to 7.67 (SD = 1.33) for the delivery system design dimension*.* The overall mean baseline ACIC-S score in the intervention group was 6.45 (SD = 1.32), indicating advanced support for integrated care for frail community-dwelling older adults. At T0, the mean overall ACIC-S score was significantly higher in the intervention group than in the control group (*p* < 0.05). The mean scores for the healthcare organization and delivery system design dimensions were also significantly higher in the intervention group than in the control group (6.92 (SD = 1.57) vs. 3.78 (SD = 2.31) and 7.67 (SD = 1.33) vs. 5.24 (SD = 2.07), respectively; both *p* < 0.001). At T1, independent samples *t*-tests showed that the mean overall ACIC-S score and scores for all six dimensions were significantly higher in the intervention group than in the control group (Table 3). We also checked the results without the five additional respondents in the control group at T1, but this revealed the same picture. Also paired analyses revealed similar findings.

**Table 3:** Quality of primary care as perceived by healthcare professionals at baseline (T0) and follow-up (T1)

ACIC-S dimension	Control group T0 *n* = 18^a^	Intervention group T0 † *n* = 57^b^	Control group T1 *n* = 23^c^	Intervention group T1 † *n* = 55^d^
	mean (SD)	mean (SD)	mean (SD)	mean (SD)
Healthcare organization	3.78 (2.31)	6.92 (1.57)**	4.85 (2.43)	6.96 (1.33)*
Community linkages	5.50 (1.70)	6.46 (1.83)	5.31 (2.17)	7.55 (1.32)**
Self-management support	5.47 (2.03)	6.03 (1.86)	4.80 (1.83)	7.03 (1.80)**
Decision support	5.07 (1.84)	5.54 (1.68)	3.98 (1.72)	5.47 (1.77)*
Delivery system design	5.24 (2.07)	7.67 (1.33)**	6.22 (2.13)	7.75 (1.65)*
Clinical information systems	6.18 (2.28)	6.10 (2.18)	4.95 (2.39)	7.01 (1.33)*
Total^e^	5.26 (1.61)	6.45 (1.32)*	5.05 (1.74)	6.98 (1.04)**

^a^0–2 missing values; ^b^0–4 missing values; ^c^0–2 missing values; ^d^0–3 missing values; ^e^range, 0–11; † Intervention group compared with control group at T0 and at T1; *ACIC-S* Assessment of Chronic Illness Care Short version, *SD* standard deviation. *p < 0.05 (two-tailed); **p < 0.001 (two-tailed); independent-samples *t*-test

### Association between interventions implemented and perceived quality of primary care

Our study results show that proactive, integrated care for frail older persons following the FFF approach  is associated with better quality of primary care. The number of interventions implemented was associated positively with ACIC-S scores at T1 (*r* = 0.56, *p* < 0.05), indicating that primary care that is congruent with (dimensions of) the CCM was of higher quality, as perceived by healthcare professionals at T1.

### Challenges related to the implementation and embedding of the FFF approach, as experienced by GPs

Although the FFF approach seems to be promising in terms of improving the quality of primary care as perceived by healthcare professionals, GPs of the intervention group identified several challenges that may hamper its sustainability and spread. The implementation and embedding of the FFF approach in GP practices requires several organizational preconditions. The identification of possible challenges experienced by the GPs is important to achieve a successful and sustainable transformation of care delivery, and to continue quality improvement in the primary care setting. Based on face-to-face interviews with the GPs, two (possible) important challenges were identified. First, the majority of GPs explained that structural financing and manpower are necessary to continue implementation of the FFF approach in the long term: “If this [the FFF approach] becomes routine care delivery, […] available means should not become unattainable, so that we have to figure it out for ourselves.” Second, GPs indicated that a facilitating information and communication technology (ICT) system is essential for accurate, uniform, and joint communication and reporting. All GPs used GP and chain information systems, which enables the exchange of information among different care disciplines. The chain information system includes disease-specific modules (e.g., for chronic obstructive pulmonary disease and diabetes care). Four GPs indicated that they implemented this system with a multi-disease module for the care of frail older patients, which can facilitate, for example, uniform reporting of individualized care plans and communications related to multidisciplinary consultations and frailty screening. However, the other seven GPs explained they had not yet implemented this module and that they experienced insufficient integration among the various databases: “I am convinced that when one would have a collective electronic platform, coordination would become even better. This can be a problem at the moment. You have to do so many things through different channels.” The aim, however, is to implement the chain information system with a module for the care of frail older patients in all GP practices that work according to the FFF approach. Other possible challenges mentioned by GPs include investment in integrated networks of involved professionals, close collaboration with specialists working at the hospital, time investment by involved professionals, and the need to plan all activities related to the FFF approach: “It is crucial to plan. At the end of each multidisciplinary consultation we plan a new appointment together. I believe that if you do not do this, we will lose ground. We should follow-up on our intended actions.”

## Discussion

The CCM incorporates important elements of healthcare systems that promote high-quality primary care delivery [17, 19, 20, 33]. The aims of our study were to increase our knowledge of the use of the CCM in primary care and to assess the quality of proactive, integrated primary care for frail community-dwelling older adults. The first study objective was to assess the implementation of interventions in the six areas of system redesign described in the CCM. Congruency of care with (elements of) the CCM in intervention GP practices that implemented the FFF approach and control GP practices delivering primary care as usual was assessed. We found that intervention GP practices implemented significantly more interventions in line with CCM dimensions on average, compared with control GP practices. The second objective was to investigate the quality of primary care as perceived by healthcare professionals in the intervention and control groups. To address this objective and gain a deeper understanding of experiences with the FFF approach, we conducted a longitudinal survey study among all involved healthcare professionals and qualitative interviews with GPs from the intervention practices. At T0, mean ACIC-S scores for the healthcare organization and delivery system design dimensions were significantly higher in the intervention group than in the control group. Consequently, the overall mean ACIC-S score was significantly higher in the intervention group than in the control group at T0. The baseline perception of higher-quality care by professionals in the intervention practices can be explained by the timing of baseline measurement. In the autumn of 2014, GP practices in the intervention group had already begun to implement elements of the FFF approach, and the majority of practices received financing for these measures via reimbursement regulations related to primary care for frail older patients. Moreover, they had already met several important preconditions, such as organizational goals and improvement strategies related to care for frail older persons. At T0, the majority of intervention GP practices was screening for frailty and holding multidisciplinary meetings. In the FFF approach, GPs select potentially frail adults in the community for screening during planned visits, and the screening results are then discussed during multidisciplinary consultations. These (partially) implemented elements of the FFF approach fall under the healthcare organization and delivery system design CCM dimensions, which may explain the higher baseline scores for these two dimensions in the intervention group. One year later, all ACIC-S scores were significantly higher in the intervention group than in the control group. Within the intervention group, professionals perceived significant improvements in the overall quality of care delivery (ACIC-S), as well as in the community linkages, self-management support, and clinical information systems dimensions, over time.

We also found that the number of interventions implemented was associated positively with the quality of primary care as perceived by healthcare professionals at T1. This finding indicates that primary care for frail older persons that is congruent with (dimensions of) the CCM is associated with better quality of primary care as perceived by healthcare professionals at the follow-up measurement.

### Motives, differences, and challenges

The main motives of GPs in the intervention practices to implement the FFF approach were the aging of the population and the need to anticipate on current transformations in the primary healthcare sector. In the Netherlands, and in many other western countries, primary care delivery is challenged by the aging of populations and the increased demand for care [12]. The Dutch government’s reforms in long-term care delivery intend to facilitate the tendency whereby older adults live independently in the community for as long as possible and access to long-term care facilities is limited [34]. Care for older persons is increasingly being delivered in the primary healthcare setting by GP practices [11], which requires the redesign of primary care delivery for frail community-dwelling older patients. GPs in the intervention group considered proactive care delivery and multidisciplinary collaboration to be essential. GPs reported considerable differences among intervention practices with respect to the implementation and execution of (elements of) the FFF approach, including proactive screening, multidisciplinary consultation, and guidelines for patient follow-up. Identification of these differences is important in determining, for example, the quality of proactive integrated care program implementation [35]. Important challenges related to the implementation and embedding of the FFF approach, as perceived by GPs, were structural financing and manpower, and access to a facilitating ICT system. The latter should include a multi-disease module for the care of frail older patients.

### Strengths and limitations

An important strength of our study was the use of a control group, which enabled us to comparatively assess the quality of care delivery and changes over time between practices providing primary care as usual and those following the FFF approach. Moreover, we used a mixed-methods design, which enabled us to gain better insight into and understanding of the implementation of (elements of) a complex proactive, integrated care approach based on the CCM and (changes in) quality of care.

The study has several limitations. First, we examined the quality of primary care as perceived by healthcare professionals. Further longitudinal research is necessary to examine the quality of primary care as experienced by frail community-dwelling older persons. Research on chronically ill patients has shown that the quality of care delivery as perceived by healthcare professionals predicted more positive experiences of patients with care delivery [36]. Moreover, the effects of the FFF approach on important patient outcomes, such as the well-being of frail older persons, service use, and associated costs, should be examined in future research. Second, healthcare professionals in the control and intervention groups showed considerable variability in occupational background and educational level. Multidisciplinary work is a core element of the FFF approach, which explains the systematic involvement of professionals in certain disciplines (e.g., elderly care physicians) in intervention, but not control, GP practices. Third, the implementation of interventions is a continuous process. As a result of national transformations in the primary healthcare sector in the Netherlands, the control GP practices were also in the process of implementing several interventions, such as medication reviews, systematic follow-up of older patients, and meetings of professionals from different disciplines to exchange information. Developments in the primary care setting and the implementation of interventions in GP practices should be monitored in the future to observe possible further improvement. Finally, we measured quality of primary care using the ACIC-S instrument, which earlier research shows is one of the available instruments which can be used to assess quality of primary care [37]. The ACIC-S measures the six dimensions of the CCM (the community, the healthcare system, self-management support, delivery system design, decision support and clinical information systems) which are needed to support frail older people and people with chronic diseases in the primary care setting. Others defined primary care by four main characteristics: comprehensive, coordinated, continuous, and accessible care and identified the Primary Care Assessment Tool (PCAT) as the best available instrument to assess such primary care features. Although both instruments clearly measure overlapping concepts and are both used regularly to assess quality of primary care [37] use of other instruments, however, may have yielded other findings.

## Conclusions

The present study showed that the FFF approach can have positive effects on the quality of primary care delivery to frail older persons, as perceived by healthcare professionals. In times of population aging and increased pressures on primary healthcare systems, proactive integrated care delivery for community-dwelling frail older persons, such as that based on the FFF approach, can be introduced to improve the perceived quality of primary care.

## Abbreviations

ACIC-S: Assessment of chronic illness care short version
CCM: Chronic care model
COPD: Chronic obstructive pulmonary disease
FFF: Finding and follow-up of frail older persons
GP: General practitioner
ICT: Information and communication technology
SD: Standard deviation
